# Re-Audit of Physical Health Equipment Available at the Mount Old Age Psychiatric Hospital

**DOI:** 10.1192/bjo.2025.10655

**Published:** 2025-06-20

**Authors:** Rosanna Pope, Megan Sheridan

**Affiliations:** Leeds and York Partnership Foundation Trust, Leeds, United Kingdom

## Abstract

**Aims:** The purpose of this audit was to re-audit (second cycle) the availability of physical health equipment on the psychiatric wards at the Mount psychiatric hospital. This was a second cycle of a previous audit performed in November 2023 to assess whether the previous recommendations had been successful in improving compliance with the 2022 CQC physical health recommended equipment list and equipment required to meet NICE guidelines for antipsychotic medication monitoring.

**Methods:** A stock check was performed of the physical health equipment available on the 4 old age psychiatric wards. This was against the items recommended in the 2020 CQC physical health guidance and key equipment required for basic investigations for monitoring of psychiatric medications, e.g. ECG and blood samples. The same criteria were used in the first cycle of this audit (completed by a different author), due to similarities in audit reference material, so this is a re-audit of the same checklist items.

Data was collected from all 4 old-age wards at the Mount on two separate occasions. Data from wards 1–3 were collected on 10/01/2025 and data from ward 4 were collected on 20/01/2025. This difference in date of data collection was due to staffing constraints.

**Results:** Overall, there was a lack of equipment across all four wards, with the percentage of recommended equipment that was not available ranging from 17.5–35%. There were 4 items that were missing across all 4 wards: Alcometer, Snellen chart, BMI chart, Tuning fork.

In addition to items that were lacking, as seen in item 2, there were several items that were either not working or expired. This includes several blood bottles and urinalysis sticks that are essential for basic monitoring. In terms of items that were not working, the only available otoscope and ophthalmoscope in the hospital was not functioning.

The variability in ECG machine function on all 4 wards means that QT interval monitoring cannot be performed reliably.

**Conclusion:** Overall, the results of the audit have shown that none of the wards at the Mount have all the necessary equipment required for adequate physical health care for psychiatric inpatients. This means that we are unable to provide adequate physical health care to psychiatric inpatients. Additionally, when compared with the results of the previous cycle of this audit there have not been significant improvements. Therefore, more clear-cut improvements are going to be required.

